# Monitoring Early Glycolytic Flux Alterations Following Radiotherapy in Cancer and Immune Cells: Hyperpolarized Carbon-13 Magnetic Resonance Imaging Study

**DOI:** 10.3390/metabo11080518

**Published:** 2021-08-06

**Authors:** Ying-Chieh Lai, Ching-Yi Hsieh, Kuan-Ying Lu, Cheng-Hsuan Sung, Hung-Yao Ho, Mei-Ling Cheng, Albert P. Chen, Shu-Hang Ng, Fang-Hsin Chen, Gigin Lin

**Affiliations:** 1 Department of Medical Imaging and Intervention, Chang Gung Memorial Hospital at Linkou, Taoyuan 333, Taiwan; cappolya@gmail.com (Y.-C.L.); fantacy52317@gmail.com (K.-Y.L.); shuhangng@gmail.com (S.-H.N.); 2 Clinical Metabolomics Core Laboratory, Chang Gung Memorial Hospital at Linkou, Taoyuan 333, Taiwan; chris11182016@gmail.com (C.-Y.H.); hoh01@mail.cgu.edu.tw (H.-Y.H.); chengm@mail.cgu.edu.tw (M.-L.C.); 3 Medical Imaging Research Center, Institute for Radiological Research, Chang Gung University, Taoyuan 333, Taiwan; a0927001062@gmail.com; 4 Department of Medical Imaging and Radiological Sciences, Chang Gung University, Taoyuan 333, Taiwan; 5 Graduate Institute of Biomedical Sciences, College of Medicine, Chang Gung University, Taoyuan 333, Taiwan; 6 Metabolomics Core Laboratory, Healthy Aging Research Center, Chang Gung University, Taoyuan 333, Taiwan; 7 Department of Medical Biotechnology and Laboratory Science, College of Medicine, Chang Gung University, Taoyuan 333, Taiwan; 8 Department of Biomedical Sciences, College of Medicine, Chang Gung University, Taoyuan 333, Taiwan; 9 GE Healthcare, Toronto, ON M5V 3Y3, Canada; Albert.Chen@ge.com; 10 Radiation Biology Research Center, Institute for Radiological Research, Chang Gung University, Taoyuan 333, Taiwan; 11 Department of Radiation Oncology, Chang Gung Memorial Hospital at Linkou, Taoyuan 333, Taiwan

**Keywords:** cancer metabolism, dynamic nuclear polarization, glycolysis, immune system, magnetic resonance imaging, radiation

## Abstract

Alterations in metabolism following radiotherapy affect therapeutic efficacy, although the mechanism underlying such alterations is unclear. A new imaging technique—named dynamic nuclear polarization (DNP) carbon-13 magnetic resonance imaging (MRI)—probes the glycolytic flux in a real-time, dynamic manner. The [1-^13^C]pyruvate is transported by the monocarboxylate transporter (MCT) into cells and converted into [1-^13^C]lactate by lactate dehydrogenase (LDH). To capture the early glycolytic alterations in the irradiated cancer and immune cells, we designed a preliminary DNP ^13^C-MRI study by using hyperpolarized [1-^13^C]pyruvate to study human FaDu squamous carcinoma cells, HMC3 microglial cells, and THP-1 monocytes before and after irradiation. The pyruvate-to-lactate conversion rate (*k*_PL_ [Pyr.]) calculated by kinetic modeling was used to evaluate the metabolic alterations. Western blotting was performed to assess the expressions of LDHA, LDHB, MCT1, and MCT4 proteins. Following irradiation, the pyruvate-to-lactate conversion rates on DNP ^13^C-MRI were significantly decreased in the FaDu and the HMC3 cells but increased in the THP-1 cells. Western blot analysis confirmed the similar trends in LDHA and LDHB expression levels. In conclusion, DNP ^13^C-MRI non-invasively captured the different glycolytic alterations among cancer and immune systems in response to irradiation, implying its potential for clinical use in the future.

## 1. Introduction

Radiotherapy is an important treatment for various cancer types. The ionizing radiation kills cancer cells directly by damaging DNA or indirectly by producing reactive oxygen species, which cause DNA breaks [1]. Although ionizing radiation is an effective anticancer modality, tumor cells may acquire resistance, leading to treatment failure. The metabolic reprogramming of cancer and stromal cells in the tumor microenvironment is an important mechanism of radioresistance [2,3,4]. In cells under stress, glucose metabolism is modified to facilitate energy production and anabolism. This metabolic alteration plays a major role in not only tumor progression, but also tumor resistance to chemotherapy and radiotherapy [2,3,4].

The enhanced cellular utility of glucose, termed the Warburg effect, is a metabolic alteration that takes place to satisfy the energy needs of cancer cells [5]. Pyruvate, the product of glycolysis, is reduced to lactate by lactate dehydrogenase (LDH), while lactate is further used as a metabolic shuttle to generate more energy [6]. The upregulation of key molecules and products in the multiple steps of the Warburg effect, including GLUT1 transporter, LDH isoenzymes, and lactate, are associated with radioresistance [7,8,9]. These molecules serve as biomarkers for radioresistance or targets for new anticancer agents [3]. The expression levels of these molecules are indirect indicators of the Warburg effect; however, clinically applicable in vivo biomarkers remain unestablished.

Although the Warburg effect was first described in cancer cells, immune cells may also undergo substantial metabolic reprogramming during cancer treatment [10,11]. Immune cells in the tumor microenvironment may constitute up to 50% of the tumor mass and play an essential role in tumor metabolism [12]. Tumor-associated immune cells are recruited from peripheral blood cells (e.g., circulating monocytes) or derived from locally self-maintained progenitor cells (e.g., microglia in the brain) [13,14]; however, the metabolic interaction between cancer cells and tumor-associated immune cells is unclear [15].

**Figure 1:**
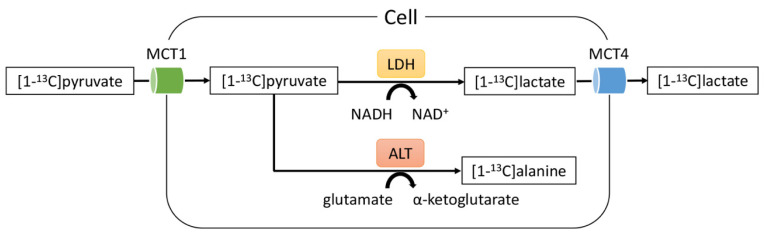
Diagram of the metabolic fates of [1-^13^C]pyruvate that were detected by hyperpolarized ^13^C-MR spectroscopy in this study. Note—MCT, monocarboxylate transporter; LDH, lactate dehydrogenase; ALT, alanine transaminase.

Hyperpolarized carbon-13 magnetic resonance imaging (^13^C-MRI) is a new non-invasive, real-time imaging technique used for detecting the glycolytic flux in cells and tissues both in vivo and in vitro [16,17]. Dynamic nuclear polarization (DNP) aims to considerably increase the polarization of solid-state compounds under an extremely low temperature and a high magnetic field, which is followed by rapid liquid dissolution [16,18]. The technique increases the signal-to-noise ratio of ^13^C-labeled probes by up to 50,000-fold, which is adequate for generating ^13^C spectra through magnetic resonance (MR) spectroscopic imaging [18]. The monocarboxylate transporter (MCT) in the cellular membrane enables the uptake of [1-^13^C]pyruvate, which is converted into [1-^13^C]lactate by lactate dehydrogenase (LDH), or to a lesser extent into [1-^13^C]alanine by alanine aminotransferase (ALT) in the cytosol (Figure 1).

Studies have demonstrated that DNP ^13^C-MRI may be a vital approach for evaluating tumor responses to irradiation in tumor models [19,20]; however, the measured ^13^C signal from the tumor mass is possibly contributed by both cancer and immune cells because tumor-associated immune cells may constitute a major proportion of the tumor mass. We hypothesized that in response to irradiation, cancer and immune cells may have different alterations in glycolytic flux. To understand the potential contributions of the overall glycolytic activities in tumors in response to irradiation, we designed a DNP ^13^C-MRI study by using [1-^13^C]pyruvate to probe cancer and immune cell lines. Western blot analysis was used to determine the expressions of LDH and MCT proteins. The DNP ^13^C-MRI revealed the different glycolytic alterations among cancer and immune systems in response to irradiation, implying its potential use in clinical settings.

## 2. Results

### 2.1. Cancer and Immune Cells

Cell suspensions, including one cancer and two immune cell lines, were included in this study. Human FaDu squamous carcinoma from hypopharyngeal cancer was selected for the cancer cell model, because head and neck cancers are commonly treated with radiotherapy. To evaluate the tumor-associated immune cells following irradiation, HMC3 microglial cells (specialized macrophages in the brain) were used to represent the tissue-resident immune cells, while THP-1 monocytes were used to represent the immune cells recruited from peripheral blood cells. The three cell lines were split into samples that were or were not subjected to 15 Gy X-ray irradiation. After 30–79 min (mean, 53 min) following irradiation, the samples were analyzed through DNP ^13^C-MRI to evaluate their glycolytic activity and through Western blot to determine the expressions of related metabolic enzymes and transporters.

### 2.2. Glycolytic Flux Alterations Following Radiotherapy in Cancer and Immune Cells

In the cancer and immune cells, the conversion of hyperpolarized [1-^13^C]pyruvate to [1-^13^C]lactate was probed using DNP ^13^C-MRI. The intensities of the labeled pyruvate and lactate from ^13^C-MR spectra were fit to a two-site exchange model. The forward (pyruvate-to-lactate) and backward (lactate-to-pyruvate) reactions and the non-recoverable hyperpolarized ^13^C signal loss due to spin-lattice relaxation and applied excitation radiofrequency pulses were included in the coupled equations as follows:

(1) $$\frac{dP\left(t\right)}{dt}=k_{\mathrm{LP}}L\left(t\right)-k_{\mathrm{PL}}P\left(t\right)-\rho P\left(t\right)$$

(2) $$\frac{dL\left(t\right)}{\mathrm{dt}}=k_{\mathrm{PL}}P\left(t\right)-k_{\mathrm{LP}}L\left(t\right)-\rho L\left(t\right)$$

In Equations (1) and (2), *P* and *L* are the relative pyruvate and lactate signal intensities, respectively; *k*_PL_ and *k*_LP_ are the forward and backward conversion rate constants, respectively. The solutions of *P*(*t*) and *L*(*t*) were solved numerically. The conversion rate constants, *k*_PL_ and *k*_LP_, were fitted into these solutions and data. The relaxivity rates of the labeled pyruvate and lactate (*ρP* and *ρL*, respectively) were assumed to be the same in this model, given in the following equation:

(3) $$\rho =\frac{1}{T_{1\;\mathrm{eff}}}=\frac{1}{T_{1}}-\frac{1}{t_{R}}\ln\left(\cos\theta \right)\;$$

**Figure 2:**
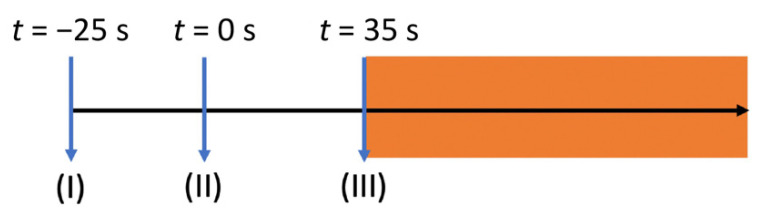
A schematic plot of the delivery timeline of hyperpolarized [1-^13^C]pyruvate. Three stages described the delivery steps: (**I**) dissolution and export of hyperpolarized [1-^13^C]pyruvate from the polarizer; (**II**) mixing of hyperpolarized [1-^13^C]pyruvate with the cell suspensions; (**III**) start of ^13^C-MR spectroscopy acquisition. Step (**II**) was defined as “*t* = 0”.

In Equation (3), *T*_1_ is the relaxation time for the metabolites in medium, *t*_R_ is the repetition time, and *θ* is the flip angle. The timeline used for kinetic modeling is presented in Figure 2.

**Figure 3:**
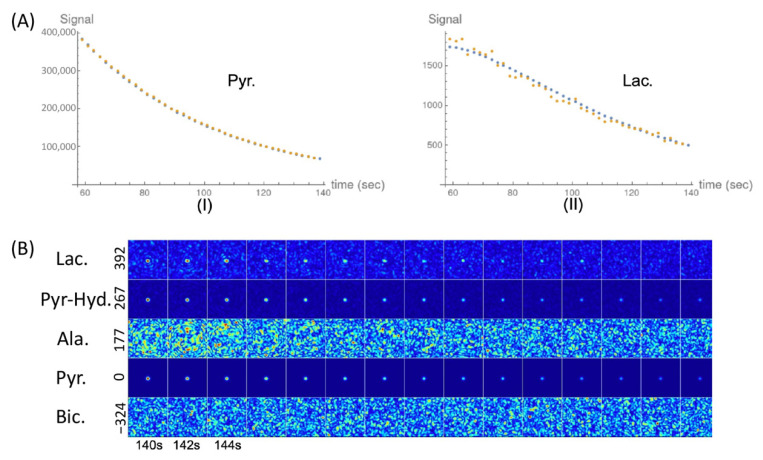
Representative data from an in vitro study. (**A**) Orange dots represent the data for [1-^13^C]pyruvate (**I**) and [1-^13^C]lactate (**II**) obtained from the spectroscopy-based ^13^C-MRI acquisition. Blue dots represent best fit lines calculated by kinetic modeling. (**B**) Imaging-based ^13^C-MRI acquisition of the investigated metabolites displayed at a temporal resolution of 2 s. Each row represents one metabolite: lactate (chemical shift, 392 Hz), pyruvate–hydrate (267 Hz), alanine (177 Hz), pyruvate (0 Hz), and bicarbonate (−324 Hz). Hyperpolarized ^13^C signals of lactate, pyruvate–hydrate, and pyruvate were visually observed.

**Figure 4:**
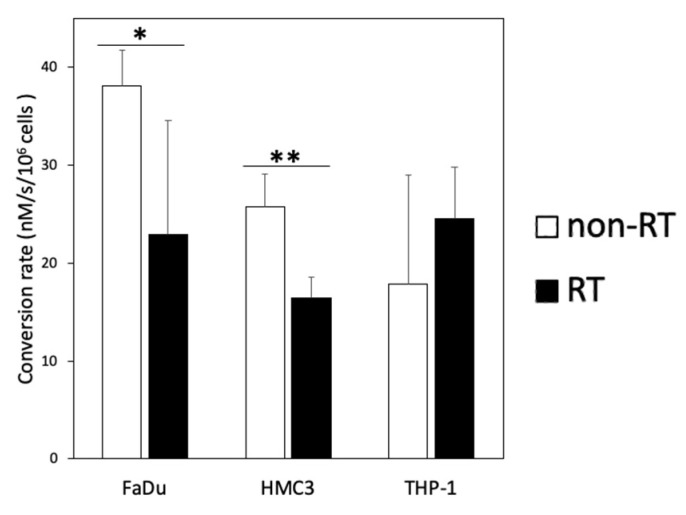
Comparison of the conversion rates (*k*_PL_ [Pyr.]) of hyperpolarized [1-^13^C]pyruvate to [1-^13^C]lactate between the non-irradiated (non-RT) and irradiated (RT) FaDu, HMC3, and THP-1 cells (* *p* < 0.05; ** *p* < 0.01). Note—*k*_PL_ [Pyr.] unit: nM/s/10^6^ cells.

Representative ^13^C-MR spectra and images of the cell experiments are presented in Figure 3. The data for the pyruvate-to-lactate conversion rate (*k*_PL_ [Pyr.]) obtained through kinetic modeling are summarized in Table 1. In the non-irradiated cell experiments, the pyruvate-to-lactate conversion rates were highest in the FaDu cancer cells, followed by the HMC3 microglial cells, then they were relatively lower in the THP-1 monocytes. In the irradiated cell experiments, the irradiated FaDu cancer cells showed a significant decrease in the pyruvate-to-lactate conversion rate from 38.1 ± 3.6 to 22.9 ± 11.6 nM/s/10^6^ cells (mean ± standard deviation, *p* = 0.049). The irradiated HMC3 microglial cells also showed a significant decrease in the pyruvate-to-lactate conversion rate from 25.7 ± 3.4 to 16.4 ± 2.2 nM/s/10^6^ cells (*p* = 0.008). In contrast, the irradiated THP-1 monocytes showed an increase in the pyruvate-to-lactate conversion rate from 17.9 ± 11.1 to 24.6 ± 5.2 nM/s/10^6^ cells, albeit not statistically significant (*p* = 0.199). Moreover, [1-^13^C]alanine was observed in both irradiated and non-irradiated HMC3 microglial cell experiments. In short, following irradiation, the pyruvate-to-lactate conversion rates were decreased in the FaDu cancer cells and the HMC3 microglial cells but increased in the THP-1 monocytes (Figure 4).

### 2.3. The Changes of LDH Corresponding to the Changes of k_PL_ [Pyr.] on DNP ^13^C-MRI

**Figure 5:**
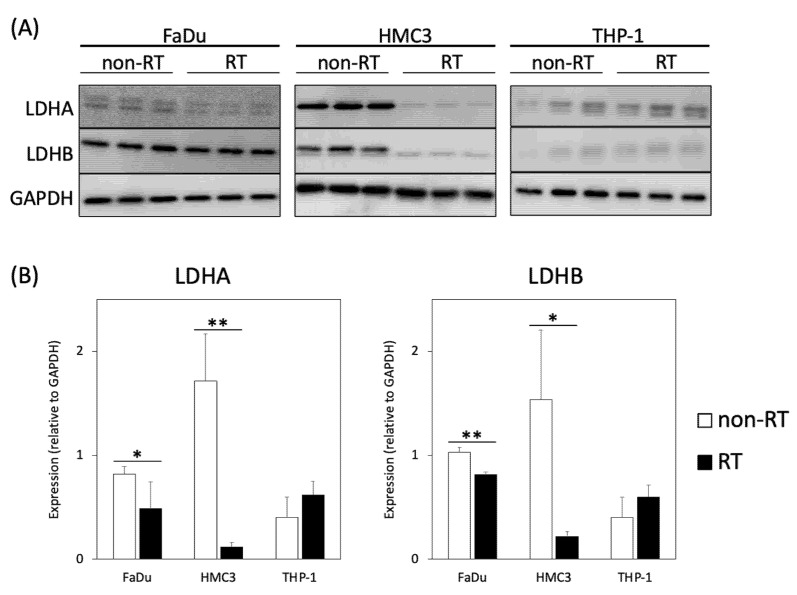
Analysis of the expressions of LDHA and LDHB in the non-irradiated (non-RT) and irradiated (RT) cancer and immune cells. (**A**) Western blot analyses of LDHA and LDHB in FaDu, HMC3, and THP-1 cells. GAPDH was used as a loading control. (**B**) Comparison of the expressions of LDHA and LDHB between the non-irradiated and irradiated cells (* *p* < 0.05; ** *p* < 0.01). Note—LDH, lactate dehydrogenase; GAPDH, glyceraldehyde-3-phosphate dehydrogenase.

Since the conversions between hyperpolarized [1-^13^C]pyruvate and [1-^13^C]lactate are mediated by LDHA (forward reaction) and LDHB (backward reaction), Western blot was performed to evaluate the expression of these key metabolic proteins before and after irradiation (Figure 5a). In the non-irradiated cell experiments, the expression levels of LDHA and LDHB were highest in the HMC3 microglial cells, followed by the FaDu cancer cells, then they relatively lower in the THP-1 monocytes. Regarding the irradiated cell experiments, the changes in the expressions of LDHA and LDHB are presented in Figure 5b. The irradiated FaDu cancer cells showed significantly decreased expression levels of LDHA and LDHB (*p* = 0.049 and 0.001, respectively). The irradiated HMC3 microglial cells also showed significantly decreased expression levels of LDHA and LDHB (*p* = 0.002 and 0.014, respectively). In contrast, the irradiated THP-1 monocytes showed increased expression levels of LDHA and LDHB, albeit not statistically significant (*p* = 0.092 and 0.107, respectively). Following irradiation, the decreased expression levels of LDHA and LDHB in the FaDu cancer cells and the HMC3 microglial cells corresponded to the decreased pyruvate-to-lactate flux on DNP ^13^C-MRI. Moreover, the upregulated LDHA and LDHB expression levels in the irradiated THP-1 monocytes also reflected the enhanced pyruvate-to-lactate flux on DNP ^13^C-MRI. On the other hand, the expression levels of the transmembrane transporters, including MCT1 (influx of pyruvate) and MCT4 (efflux of lactate), were also analyzed (Figure S1). In the FaDu cancer cells, the expression levels of MCT1 and MCT4 were not detectable in either non-irradiated or irradiated cells. In the irradiated HMC3 microglial cells, there was no significant change in the expression of MCT1 or MCT4 (*p* = 0.301 and 0.159, respectively). The irradiated THP-1 monocytes showed significantly increased expression of MCT4 (*p* = 0.009), while there was no significant change in the expression of MCT1 (*p* = 0.173).

## 3. Discussion

The present study demonstrates the potential for monitoring early metabolic alterations in both cancer and immune cells following radiation treatment by using hyperpolarized ^13^C-MRI. We demonstrated that cancer cells (human FaDu squamous carcinoma) and immune cells (HMC3 microglial cells and THP-1 monocytes) had distinct metabolic responses to ionizing radiation. MR spectroscopy with a ^13^C-labeled substrate was applied to study cancer cell metabolism in preclinical settings [21,22]. We furthered the knowledge of metabolic alterations resulting from not only cancer cells but also immune cells in the tumor microenvironment [13].

The pyruvate-to-lactate conversion was investigated as a surrogate to monitor metabolic response. In a DNP ^13^C-MRI study evaluating the irradiation effects on human breast cancer, a decreased lactate-to-pyruvate ratio was captured in a cell model 96 h after irradiation [20]. Accordingly, in human squamous carcinoma, we postulated that radiation response could be observed even earlier by using the decreased pyruvate-to-lactate conversion rate on DNP ^13^C-MRI. Radiation-induced cell death depends on the cytotoxic effects of free radicals, while pyruvate is an effective free radical scavenger that neutralizes the reactive oxygen species produced by ionizing radiation [23]. The pyruvate-to-lactate conversion determined by DNP ^13^C-MRI was utilized as an indirect measurement of the tumor-reducing potential; that is, the ability to reduce radiation-induced oxidative stress [19]. Additionally, cancers can switch between glycolysis and oxidative phosphorylation to meet their energy needs. Although enhanced glycolysis by converting glucose to lactate is a metabolic hallmark of cancer cells, it has been demonstrated that the irradiated cancer cells may shift from glycolysis to oxidative phosphorylation (termed the reverse Warburg effect) [24]. Moreover, radiation-induced cell death and quiescence may also contribute to decreased glycolytic flux in the irradiated cancer cells.

In the caner microenvironment, immune cells can be derived from locally self-maintained progenitor cells (e.g., HMC3 microglial cells) or recruited from peripheral blood cells (e.g., THP-1 monocytes) [13,14]. Both cell types may coexist in the tumor microenvironment and be involved in diverse functions ranging from early carcinogenesis to tumor invasion and metastasis [25,26]. Although the Warburg effect was first described in cancer cells, immune cells may also undergo glycolytic reprogramming [10,11]. Microglia cells, as resident macrophages, are triggered by irradiation [27]. Different degrees of dependance on glycolysis or oxidative phosphorylation lead to different degrees of polarization of microglia cells [28]. We observed that the HMC3 microglial cells showed downregulated glycolysis following irradiation. In microglia cells, a preference for oxidative phosphorylation over glycolysis has been linked to an anti-inflammatory phenotype [28]. Importantly, the production of alanine was observed solely in the HMC3 microglial cell experiments. The microglial cells in the human brain are highly flexible in terms of energy production and may use glutamate as a fuel for energy [29]. Glutamate and pyruvate are converted into alanine and α-ketoglutarate, then α-ketoglutarate enters the tricarboxylic acid cycle as an additional carbon supply. In addition, alanine can be a precursor for gluconeogenesis in cells with a high energy demand. On the other hand, monocytes can also switch between glycolysis and oxidative phosphorylation, displaying diverse phenotypes that contribute to pro- and anti-inflammatory immunity [30,31]. In our study, we found that the THP-1 monocytes displayed upregulated glycolysis following irradiation. A study showed that irradiation promotes an influx of circulating monocytes into the tumor microenvironment, which is related to tumor recurrence after radiotherapy [32]. During the process, enhanced glycolysis plays an essential role in the adhesion of monocytes to the vascular endothelium, which facilitates the infiltration of monocytes into the tumor [33].

In this study, our kinetic model was based on a two-site interaction between the hyperpolarized [1-^13^C]pyruvate and [1-^13^C]lactate, without considering the transmembrane transport of the metabolites. We demonstrated that following irradiation, the changes of pyruvate-to-lactate flux on DNP ^13^C-MRI reflected the changes of LDHA and LDHB expression levels on Western blots. In many DNP ^13^C-MRI studies, the pyruvate-to-lactate conversion could be attributed to LDH activity [34,35]; however, a recent study proved that the transmembrane flux of pyruvate mediated by MCT1 is the rate-limiting step [36]. To consider the transmembrane flux in cell studies, three-site models that include the extracellular compartment have been proposed; however, in line with our data, their measurements in *k*_PL_ were not significantly different from those calculated from the less complex two-site model [37].

Despite the novelty, certain limitations need to be addressed. First, our experiments were performed with cell suspensions, which may not reflect the complex metabolic interactions between cancer cells and the tumor microenvironment in the real world. The results of our study should be further validated in three-dimensional cell cultures that more closely resemble the in vivo microenvironment. Moreover, the expression levels of related glycolytic proteins should be confirmed by using immunochemical staining, not only for quantification, but also for localization. Second, a time gap existed between the manual mixing and the initiation of ^13^C-MRI acquisition; thus, the metabolic process during the first 50 s was not captured through ^13^C-MRI. Last, the complex tumor microenvironment includes not only immune cells but also endothelial cells, and their roles should be considered and warrant future investigation.

In conclusion, this preliminary study demonstrated the ability of DNP ^13^C-MRI to capture the different glycolytic alterations among cancer and immune systems following irradiation, indicating its potential use in clinical settings. The investigated cells, including cancer and immune cells, exhibited distinct glycolytic reprogramming in response to irradiation; however, our understanding of the metabolic interactions between cancer and immune cells is rather limited based on this preliminary study. Future studies are warranted to validate to role of DNP ^13^C-MRI in monitoring the early metabolic response following treatment.

## 4. Materials and Methods

### 4.1. Cell Preparation and Irradiation

Human FaDu squamous carcinoma cells, HMC3 microglial cells, and THP-1 monocytes were purchased from the American Type Culture Collection (Frederick, MD, USA) and maintained in minimum essential medium (MEM), Roswell Park Memorial Institute 1640 (RPMI 1640), and Dulbecco’s modified Eagle’s medium (DMEM) (Thermo Fisher Scientific, Waltham, MA, USA), respectively. All culture media contained 10% fetal bovine serum and 1% penicillin–streptomycin (Thermo Fisher Scientific, Waltham, MA, USA). The cells were incubated at 37 °C in a humidified 5% CO_2_ and 95% air atmosphere. They were trypsinized after phosphate-buffered saline (PBS) wash and their numbers and viability were determined using the LUNA-FL dual-fluorescence cell counter (Logos Biosystems, Anyang-si, Gyeonggi-do, Korea). The cell numbers ranged from 2.2 × 10^7^ to 9.4 × 10^7^, with cell viability being approximately 80% for each cell line. The cells were then centrifugated and resuspended in 9 mL of the mixed medium (1 mL of used medium and 8 mL of fresh medium) at a cell density of >1 × 10^7^ viable cells/mL. The pH was adjusted to 6 through the addition of 5 μL of acetic acid to the fresh medium before mixing. The cells were split into samples that were or were not subjected to 15 Gy radiation using 6-MV X-rays from a linear accelerator at a dose rate of 6 Gy/min. The time interval between the radiation treatment and ^13^C-MRI acquisition ranged from 30 to 79 min (mean, 53 min).

### 4.2. [1-13. C]Pyruvate Hyperpolarization and In Vitro Experiments

Research-grade fluid paths (RFP; GE Healthcare, Chicago, IL, USA) were filled with 35 mg of [1-^13^C]pyruvic acid doped with 15 mM electron paramagnetic agent (trityl radical AH111501; GE Healthcare, Chicago, IL, USA) and 14 g of water containing 0.1 g/L ethylenediaminetetraacetic acid (EDTA) dissolution medium. Samples were polarized using a clinical hyperpolarizer (SPINlab; GE Healthcare, Chicago, IL, USA) at a temperature of 0.8 K and a magnetic field of 5 T for an average of 180 min. Following rapid dissolution, the pyruvic acid solution was neutralized and diluted with TRIS-buffered NaOH solution to obtain approximately 5 mL of [1-^13^C]pyruvate solution at neutral pH. Next, 1 mL of the fluid containing approximately 75 mM hyperpolarized [1-^13^C]pyruvate was immediately added to 9 mL of the cell suspension in a syringe, resulting in a final pyruvate concentration range of 7.0–7.5 mM. The time intervals between the dissolution of hyperpolarized [1-^13^C]pyruvate and the start of ^13^C-MRI acquisition ranged from 62 to 77 s. The temperature of the samples was regulated at approximately 37.0 °C during MR imaging. The pH levels of the samples ranged from 5.9 to 6.4. The experiments were repeated three times for the non-irradiated and irradiated cells, including FaDu cancer cells, HMC3 microglial cells, and THP-1 monocytes.

### 4.3. Imaging Acquisition

Imaging was performed using a clinical 3T MRI system (Discovery MR750w; GE Healthcare, Chicago, IL, USA) with imaging sequences from the Multinuclear Spectroscopy (MNS) research pack. Cell suspension syringes were imaged using a 35-mm-diameter ^13^C/^1^H multinuclear transmit–receive coil (RAPID Biomedical, Rimpar, Bavaria, Germany). The ^13^C-MRI was performed using spectroscopy-based acquisition followed by imaging-based acquisition. The spectroscopy-based ^13^C data were initially acquired using a pulse-and-acquire sequence with a nominal flip angle of 10°. The imaging parameters were as follows: read bandwidth, 5000 Hz; repetition time, 2000 ms; slice thickness, 20 mm; spectral points collected, 2048; repetition, 40 times. The aforementioned procedures were immediately followed by 32 s of imaging-based acquisition using iterative decomposition with echo asymmetry and least squares estimation (IDEAL) spiral chemical shift imaging (CSI) [38]. Each excitation was followed by single-shot spiral imaging, with echo time shifting of 1.12 ms between the excitations. Seven time-shifted echoes plus a single free induction decay (FID) spectrum were acquired for each time step, with the chemical shift information from the FID spectra providing prior knowledge for the reconstruction. The temporal resolution was 2 s. Other imaging parameters were as follows: repetition time, 250 ms; slice thickness, 20 mm; flip angle, 10°; field of view, 200 mm; nominal matrix resolution, 32 × 32 points. The raw spectroscopy data were reconstructed, apodized, phase-corrected, and background-subtracted using SAGE software (GE Healthcare, Chicago, IL, USA). The data acquired using the pulse-and-acquire sequence were used for kinetic modeling.

### 4.4. Western Blot

The cells were washed in PBS and lysed in ice-cold radioimmunoprecipitation assay (RIPA) buffer (Thermo Fisher Scientific, Waltham, MA, USA) with protease inhibitor cocktail (Roche, Penzberg, Bavaria, Germany) for 5 min. The extracts were centrifuged at 14,000 g for 15 min at 4 °C. The protein concentration was determined using the bicinchoninic acid (BCA) assay (Thermo Fisher Scientific, Waltham, MA, USA). The lysates were boiled for 5 min in the presence of sodium dodecyl sulfate (SDS) sample buffer, electrophoresed by 10% dodecyl sulfate polyacrylamide gel electrophoresis (SDS–PAGE), and transferred to polyvinylidene fluoride membranes (Immun-Blot PVDF; Bio-Rad Laboratories, Irvine, CA, USA). The membranes were incubated with a blocking buffer (5% nonfat dry milk and Tris-buffered saline with 0.1% Tween 20) for 1 h at room temperature and incubated overnight at 4 °C with primary antibodies for LDHA (Rabbit Ab; Cell Signaling Technology, Danvers, MA, USA; #3582; 1:1000 dilution), LDHB (Rabbit Ab; Novus, Centennial, CO, USA; #NBP2-53421; 1:400 dilution), MCT1 (Rabbit Ab; Novus, Centennial, CO, USA; #NBP1-59656; 1:250 dilution), and MCT4 (Rabbit Ab; Novus, Centennial, CO, USA; #NBP1-81251; 1:1000 dilution). This was followed by incubation with secondary goat antirabbit IgG or goat antimouse IgG antibody conjugated to horseradish peroxidase (Thermo Fisher Scientific, Waltham, MA, USA). The bands were visualized using a super signal chemiluminescence Western blot kit (Thermo Fisher Scientific, Waltham, MA, USA). An anti-glyceraldehyde-3-phosphate dehydrogenase (anti-GAPDH) antibody was used to verify equal protein loading.

### 4.5. Statistics

All data were analyzed using PRISM (version 8.0; GraphPad Software, San Diego, CA, USA). All data are represented as means ± standard deviation. Continuous variables were compared using Student’s t-test. Two-tailed *p* < 0.05 was considered statistically significant.

## Figures and Tables

**Table 1:** Conversion rates (*k*_PL_ [Pyr.]) of hyperpolarized [1-^13^C]pyruvate to [1-^13^C]lactate in cells.

Cells	Non-RT		RT		*p*-Value *
	*k*_PL_ [Pyr.]	*R* ^2^	*k*_PL_ [Pyr.]	*R* ^2^	
FaDu					0.049
	34.1	0.821	25.6	0.963	
	41.2	0.780	10.2	0.901	
	39.0	0.948	33.0	0.923	
HMC3					0.008
	29.2 ^†^	0.827	17.6	0.298	
	22.5	0.980	13.9 ^†^	0.988	
	25.5	0.945	17.7	0.900	
THP-1					0.199
	17.2	0.996	22.6	0.993	
	29.3	0.990	30.5	0.984	
	7.2	0.928	20.6	0.746	

*R*^2^ is the coefficient of determination of each model fitting to [1-^13^C]lactate data. * Comparison of *k*_PL_ [Pyr.] between the non-RT and RT cells using Student’s *t*-test. ^†^ The [1-^13^C]alanine signal was detected in the experiment. Note—*k*_PL_ [Pyr.] unit: nM/s/10^6^ cells; non-RT, non-irradiated; RT, irradiated.

## Data Availability

The data that support the findings of this study are available from the corresponding author upon reasonable request.

## Supplementary Materials

The following are available online at https://www.mdpi.com/article/10.3390/metabo11080518/s1, Figure S1: Analysis of the expressions of MCT1 and MCT4 in the non-irradiated and irradiated cancer and immune cells.
