# Supplementary material for: Monitoring Early Glycolytic Flux Alterations Following Radiotherapy in Cancer and Immune Cells: Hyperpolarized Carbon-13 Magnetic Resonance Imaging Study
Source: Metabolites. 2021 Aug 6;11(8):518. doi: 10.3390/metabo11080518 (PMC8398834; doi:10.3390/metabo11080518)
Supplement: Supplementary file 1 [file metabolites-11-00518-s001.zip › metabolites-1260257-SI.pdf]

metabolites

MDPI

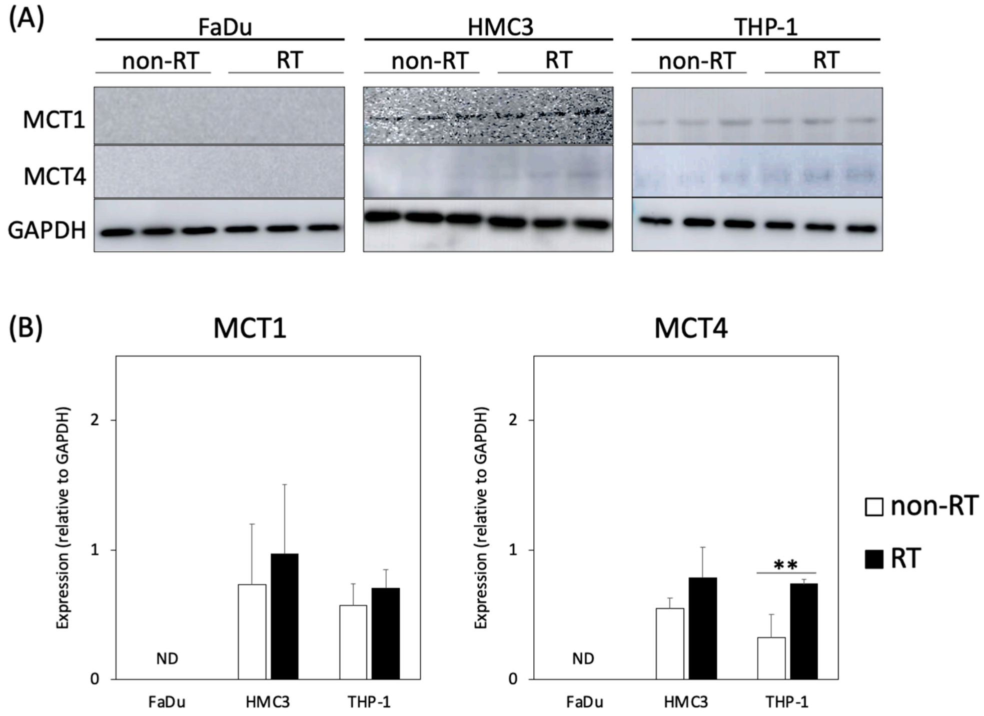

Figure S1. Analysis of the expressions of MCT1 and MCT4 in the non-irradiated (non-RT) and irradiated (RT) cancer and immune cells. (A) Western blot analyses of MCT1 and MCT4 in FaDu, HMC3, and THP-1 cells. GAPDH was used as a loading control. (B) Comparison of the expressions of MCT1 and MCT4 between the non-irradiated and irradiated cells (** $P < 0.01$). Note—MCT, monocarboxylate transporter; GAPDH, glyceraldehyde-3-phosphate dehydrogenase; ND, not detectable.
